# Identifying potential risk genes and pathways for neuropsychiatric and substance use disorders using intermediate molecular mediator information

**DOI:** 10.3389/fgene.2023.1191264

**Published:** 2023-06-21

**Authors:** Huseyin Gedik, Tan Hoang Nguyen, Roseann E. Peterson, Christos Chatzinakos, Vladimir I. Vladimirov, Brien P. Riley, Silviu-Alin Bacanu

**Affiliations:** ^1^ Integrative Life Sciences, Virginia Institute of Psychiatric and Behavioral Genetics, Virginia Commonwealth University, Richmond, VA, United States; ^2^Department of Psychiatry, Virginia Institute for Psychiatric and Behavioral Genetics, Virginia Commonwealth University, Richmond, VA, United States; ^3^ Institute for Genomics in Health, SUNY Downstate Health Sciences University, Brooklyn, NY, United States; ^4^Department of Psychiatry and Behavioral Sciences, SUNY Downstate Health Sciences University, Brooklyn, NY, United States; ^5^ Stanley Center for Psychiatric Research, Broad Institute of MIT and Harvard, Cambridge, MA, United States; ^6^Department of Psychiatry, McLean Hospital and Harvard Medical School, Belmont, MA, United States; ^7^Department of Psychiatry, College of Medicine, University of Arizona Phoenix, Phoenix, AZ, United States

**Keywords:** eQTL, pQTL, mQTL, Mendelian randomization, alcohol use disorder, bipolar disorder, schizophrenia

## Abstract

Neuropsychiatric and substance use disorders (NPSUDs) have a complex etiology that includes environmental and polygenic risk factors with significant cross-trait genetic correlations. Genome-wide association studies (GWAS) of NPSUDs yield numerous association signals. However, for most of these regions, we do not yet have a firm understanding of either the specific risk variants or the effects of these variants. Post-GWAS methods allow researchers to use GWAS summary statistics and molecular mediators (transcript, protein, and methylation abundances) infer the effect of these mediators on risk for disorders. One group of post-GWAS approaches is commonly referred to as transcriptome/proteome/methylome-wide association studies, which are abbreviated as T/P/MWAS (or collectively as XWAS). Since these approaches use biological mediators, the multiple testing burden is reduced to the number of genes (∼20,000) instead of millions of GWAS SNPs, which leads to increased signal detection. In this work, our aim is to uncover likely risk genes for NPSUDs by performing XWAS analyses in two tissues—blood and brain. First, to identify putative causal risk genes, we performed an XWAS using the Summary-data-based Mendelian randomization, which uses GWAS summary statistics, reference xQTL data, and a reference LD panel. Second, given the large comorbidities among NPSUDs and the shared cis-xQTLs between blood and the brain, we improved XWAS signal detection for underpowered analyses by performing joint concordance analyses between XWAS results i) across the two tissues and ii) across NPSUDs. All XWAS signals i) were adjusted for heterogeneity in dependent instruments (HEIDI) (non-causality) *p*-values and ii) used to test for pathway enrichment. The results suggest that there were widely shared gene/protein signals within the major histocompatibility complex region on chromosome 6 (*BTN3A2* and *C4A*) and elsewhere in the genome (*FURIN, NEK4, RERE,* and *ZDHHC5*). The identification of putative molecular genes and pathways underlying risk may offer new targets for therapeutic development. Our study revealed an enrichment of XWAS signals in vitamin D and omega-3 gene sets. So, including vitamin D and omega-3 in treatment plans may have a modest but beneficial effect on patients with bipolar disorder.

## 1 Introduction

Genome-wide association studies (GWAS) have identified numerous loci associated with neuropsychiatric and substance use disorders (NPSUDs). Furthermore, the risk loci of NPSUDs have not been fully discovered (Owen and Williams, 2021). For instance, the largest GWAS for schizophrenia (SCZ) found 287 independent loci and estimates that the common variants explain only 24% of the phenotypic variance (Trubetskoy et al., 2022). Similarly, other NPSUD GWAS yield large numbers of genome-wide significant signals (Howard et al., 2019; Nievergelt et al., 2019; Sanchez-Roige et al., 2019; Mullins et al., 2021) that capture a statistically significant but small proportion of the phenotypic variance. Since most genome-wide significant signals reside in non-protein coding genomic regions (Edwards et al., 2013), the interpretation of these GWAS findings are not straightforward. Performing post-GWAS analyses that infer associations between genes or molecular pathways and traits could significantly advance our understanding of these GWAS signals.

Associated variants are thought to influence risk through altered gene regulation, e.g., via changes in the RNA levels, protein abundance, or epigenetic markers. This assumption is supported by empirical studies of quantitative locus mapping, which found that expression quantitative trait loci (eQTL) (Ongen et al., 2017), protein QTL (pQTL) (Robins et al., 2021), and methylation QTL (mQTL) (Hannon et al., 2016) colocalize with disease-associated loci. However, while there are many well-powered GWAS scans of NPSUDs (Pardiñas et al., 2018; Demontis et al., 2019; Grove et al., 2019; Howard et al., 2019; Nievergelt et al., 2019; Watson et al., 2019; Johnson et al., 2020; Polimanti et al., 2020; Mullins et al., 2021; Trubetskoy et al., 2022), none of these studies directly assayed the transcriptome, proteome, or methylome for their cohorts.

However, researchers found ways around this assessment limitation in GWAS cohorts. They assembled large blood and brain reference molecular e/p/mQTL (henceforth denoted as xQTL) datasets that are publicly available (Sun et al., 2018; van der Wijst et al., 2020; Yang C. et al., 2021; Ferkingstad et al., 2021; Võsa et al., 2021; Zhang et al., 2021). Researchers have developed methods to integrate these molecular xQTL data and GWAS summary statistics to impute the association between phenotypes and molecular mediators (transcriptome, proteome, and methylome). Such analyses are widely referred to as transcriptome-wide association studies (TWAS), proteome-wide association studies (PWAS), and methylome-wide association studies (MWAS) (Gamazon et al., 2015; Gusev et al., 2016; Zhu et al., 2016; Barbeira et al., 2018; Barbeira et al., 2019; Hu et al., 2019; Nagpal et al., 2019; Bae et al., 2021)—henceforth collectively referred to as XWAS. Moreover, since they directly model relevant biological mediators, these approaches could identify putatively causal genes (Wainberg et al., 2019). Until recently, XWAS analyses of NPSUDs were mostly TWAS (Zhu et al., 2016; Niu et al., 2019; Hammerschlag et al., 2020; Kapoor et al., 2021). However, PWAS analyses are also increasing in number with the expanding pQTL reference data in the brain (Wingo et al., 2021; Wingo et al., 2022). In addition to the transcriptome and proteome, the methylome has also been investigated as a mediator (Perzel Mandell et al., 2021; Shen et al., 2022). Recently, MWAS yielded significant genes for NPSUDs (Sugawara et al., 2018; Aberg et al., 2020; Howard et al., 2022).

Changes in gross anatomical and cell type-specific phenotypes have been observed for NPSUDs and associated risk alleles via *in vitro* and *post-mortem* studies (Brennand et al., 2012; Schrode et al., 2019; Zhang et al., 2020). Functional genomic profiles differ by cell type (Marstrand and Storey, 2014; Buenrostro et al., 2015), and cell type composition differs across brain regions (Wang et al., 2018). In addition, different neuronal cell types have different functional profiles and different distributions across regions (Kelley et al., 2018). Genetic variants contributing to the heritability of certain NPSUDs were enriched in cis-regulatory elements that are specific to GABAergic and glutamatergic neurons (Sanchez-Priego et al., 2022). Thus, integrating cell type-specific xQTL with GWAS findings is very promising. However, due to expense and other factors, sample sizes for functional profiles in specific cell types across brain regions are still small (Spaethling et al., 2017; Bryois et al., 2022) and limited in detection power. Consequently, most functional genomics data available to support xQTL mapping studies come from bulk brain tissue (GTEx Consortium, 2020) rather than a single cell (Bryois et al., 2022) or sorted cell types (Aygün et al., 2021), and despite their limited cellular resolution, the use of bulk tissue and meta-analysis across tissues is currently still more powerful.

To conduct an XWAS, two common approaches, TWAS (Gusev et al., 2016) and PrediXcan (Gamazon et al., 2015), have been used, with both requiring pre-computing of SNP weights from xQTL datasets. To avoid LD confounding, these XWAS tools also require a subsequent fine-mapping step—e.g., TWAS-FOCUS (Mancuso et al., 2019). In contrast, Mendelian randomization (MR)-based methods (Zhu et al., 2016; Yuan et al., 2020; Zhou et al., 2020) do not require the pre-computation of SNP weights and test for inference in a two-step regression framework. Among MR-based XWAS methods, summary-data-based Mendelian randomization (SMR) is among the most commonly used methods (Zhu et al., 2016). It has the advantage of providing users with a heterogeneity in dependent instruments (HEIDI) test to filter out non-causal loci that may be just in linkage with causal signals.

There is widespread comorbidity among NPSUDs (Plana-Ripoll et al., 2019). This is in part due to shared genetic risk factors (Lee et al., 2019), e.g., as detected by the genetic correlation (r_G_) in cross-trait analyses (Brainstorm Consortium et al., 2018). Consequently, it is possible that there could be many shared XWAS signals among NPSUDs. This supports the joint analysis of NPSUDs to potentially increase detection power, especially for underpowered disorders (Turley et al., 2018; Gleason et al., 2020; Taraszka et al., 2022). Additionally, there is significant concordance of cis-eQTL and cis-mQTL effects between blood and the brain (Qi et al., 2018), and more than 70% of cis-pQTL are shared between blood and the brain (Yang C. et al., 2021). Moreover, the direction of effects across most tissues for shared eQTLs is the same (GTEx Consortium, 2015). Consequently, given the high comorbidities between traits and xQTL concordance between tissues, a joint analysis of the XWAS results from all traits and tissues would likely help uncover novel signals, especially for relatively underpowered NPSUDs and tissues (e.g., brain).

In this study, we used SMR to perform blood and brain XWAS of NPSUDs to identify potential molecular mediators for these disorders. To increase signal detection in underpowered disorders and tissue (brain), we leveraged comorbidities between NPSUD and cis-xQTL cross-tissue agreement in a joint trait/tissue concordance analysis. Subsequent analyses of XWAS signals were used to uncover putative risk loci and pathways, shedding light on the etiology of NPSUDs.

## 2 Materials and methods

### 2.1 Statistical method

**FIGURE 1:**
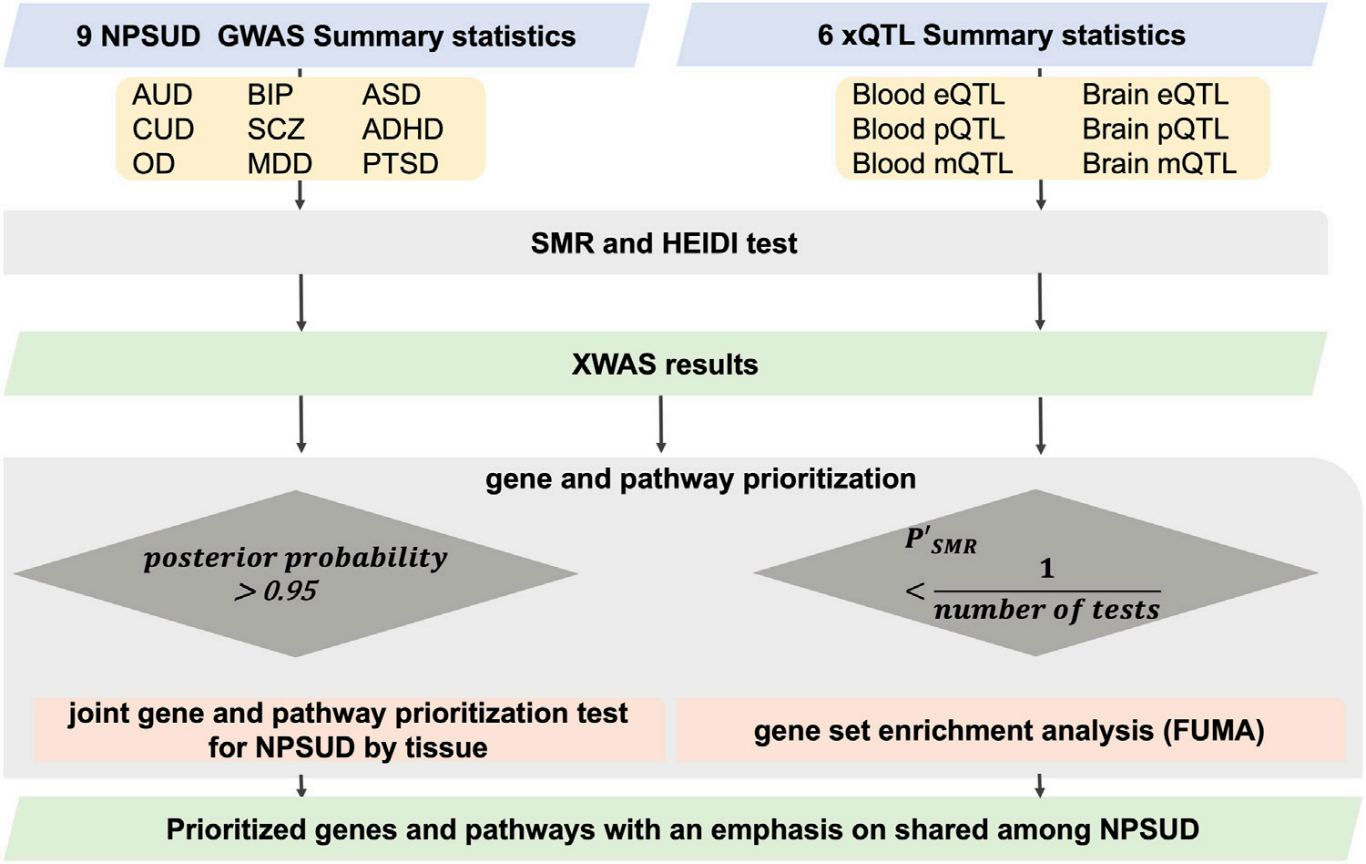
Flowchart of SMR XWAS analyses paired in both blood and brain tissues. To penalize for heterogeneity (non-causality), we employed an adjusted probe *p*-value [ $P_{SMR}^{\prime }$ = $P_{SMR}$ /min ( $P_{HEIDI}$ /0.01,1)]. For gene set enrichment analysis, we used the suggestive signals (expected to occur once per scan by chance). The Primo method was used to conduct multitrait analysis, and functional mapping and annotation (FUMA) was used for gene set enrichment analysis.

We performed univariate XWAS analyses for nine NPSUDs [attention-deficit and hyperactivity disorder (ADHD), autism spectrum disorder (ASD), alcohol use disorder (AUD), bipolar disorder (BIP), cannabis use disorder (CUD), major depressive disorder (MDD), opioid use/dependence disorder (OD), post-traumatic stress disorder (PTSD), and schizophrenia (SCZ)] (Table 1) for three XWAS paradigms (TWAS, PWAS, and MWAS) and two tissues (blood and brain). For this purpose, we used SMR (v.1.03) (Zhu et al., 2016) to infer the association between the transcriptome/proteome/methylome and NPSUDs. We performed SMR analysis for GWAS of NPSUDs (Table 1) using external xQTL reference datasets (Table 2). To prioritize genes and perform pathway analyses, we adjusted the probe (RNA/protein/CpG) SMR *p*-value ( $P_{SMR}$ ) for the HEIDI test *p*-value ( $P_{HEIDI}$ ) by combining the two *p*-values into a single one by requiring that i) $P_{SMR}$ was not penalized when $P_{HEIDI}$ was above 0.01 and ii) $P_{SMR}$ was penalized by the amount of $P_{HEIDI}$ that fell below 0.01. Consequently, we adjusted $P_{SMR}$ to $P_{SMR}^{\prime }=\frac{P_{SMR}}{\min\left(\frac{P_{HEIDI}}{0.01},1\right)}$ . We used this approach instead of filtering by $P_{HEIDI}<0.01$ because a misalignment between the GWAS cohort population and the European LD reference panel used by SMR might yield very low $P_{HEIDI}$ , e.g., the well-known *C4A* in our SCZ TWAS ( $P_{HEIDI}=5.94\times 10^{-4}$ ). Subsequently, to extend the inference to pathways, we performed a gene set enrichment analysis for suggestive ( $P_{SMR}^{\prime }<\frac{1}{number\;of\;probes}$ ) signals (Figure 1).

**TABLE 1:** Summary statistics of eight major PGC GWAS and MVP AUD GWAS.

Neuropsychiatric and substance use disorders	GWAS significant markers^a^/total markers	Study	Number of cases and controls/ancestry
Attention-deficit and hyperactivity disorder	317/8,094,095	Demontis et al. (2019)	19,099–34,194/EUR
Autism spectrum disorder	93/7,822,833	Grove et al. (2019)	18,381–27,969/EUR
Alcohol use disorder	588/6,895,251	Kranzler et al. (2019)	34,658–167,346/EUR
Bipolar disorder	3,205/7,608,184	Mullins et al. (2021)	41,917–371,549^b^/EUR
Cannabis use disorder	29/7,735,104	Johnson et al. (2020)	20,196–363,116/EUR
Major depressive disorder	4,625/7,286,335	Howard et al. (2019)	411,965–1,285,068/EUR
Opioid use/dependence disorder	0/4,571,339	Polimanti et al. (2020)	4,503–32,500/EUR
Post-traumatic stress disorder	3,434/3,875,929	Nievergelt et al. (2019)	30,000–170,000/EUR
Schizophrenia	22,344/7,585,077	Trubetskoy et al. (2022)	33,640–43,456/mostly EUR

^a^ Unpruned (based on LD) variants.

^b^ Case group includes individuals with bipolar or unipolar, and control groups include individuals without any such diagnosis.

EUR: European.

**TABLE 2:** Reference xQTL molecular datasets used for XWAS studies.

Study	Tissue	Sample size	Study	Genotype	Probe assay	Number of probes in SMR analysis
Reference eQTL data						
eQTLGen	Peripheral blood	31,684	Võsa et al. (2021)	SNP array	Expression array and RNA-seq	19,250
BrainMeta v2*	Brain cortex	2,865 (effective sample size: 2,443)	Qi et al. (2022)	SNP array/WGS	Expression array and RNA-seq	16,744
Reference pQTL data						
deCODE**	Blood plasma	35,559	Ferkingstad et al. (2021)	SNP array/WGS	SOMAscan	4,773
ROS/MAP-Banner- MSBB	dPFC + FC/dPFC/PG	366 + 70/151/135	Wingo et al. (2022)	WGS	TMT isobaric labeling MS	9,346
Reference mQTL data						
Blood mQTL***	Peripheral blood	1,980	McRae et al. (2018); Wu et al. (2018)	SNP array	Illumina HumanMethylation450 array	94,338
Brain mQTL****	dPFC/fetal brain	1,160	Qi et al. (2018)	SNP array	Illumina HumanMethylation450 array	436,077

SOMAScan: slow off-rate modified aptamer scan; WGS: whole-genome sequencing; ROS: Religious Orders Study; MAP: Rush Memory and Aging Project; MSBB: Mount Sinai NIH NeuroBioBank; dPFC: dorsolateral prefrontal cortex; FC: frontal cortex; PG: parahippocampal gyrus; TMT: tandem mass tag; MS: mass spectrometry.

^a^ These data are a meta-analysis of the GTEx brain, CMC, and ROS/MAP by using MeCS (Qi et al., 2018).

^b^ One group of participants is from deCODE, and the second group is from the Icelandic Cancer Project.

^c^ Meta-analysis of mQTL data from two independent cohorts: Brisbane System Genetics (BSGS) and Lothian Birth Cohorts (LBC).

^d^ Meta-analysis of mQTL data from three independent cohorts [Hanon et al., Jaffe et al., and ROS/MAP (Hannon et al., 2016; Jaffe et al., 2016; Ng et al., 2017)] by MeCS.

### 2.2 Parameters for SMR-based XWAS analyses

SMR analyses were performed only for cis-xQTLs (SNPs with *p*-value < 5 × 10^−8^ within 2 Mbp of the probe). We also used the default maximum (20) and minimum (3) number of xQTLs selected for the HEIDI test. We set the significance threshold as < 1.57 × 10^−3^ for the HEIDI test *p*-values and the mismatch of minimum allele frequency among input files as < 15%. For the HEIDI test, SNPs with LD > 0.9 and <0.05 with the top associated xQTL SNPs were pruned. In case-control studies, we log-transformed the odds ratio as suggested by the SMR analysis guidelines (https://yanglab.westlake.edu.cn/software/smr/#SMR&HEIDIanalysis, accessed on 3 August 2022).

### 2.3 Neuropsychiatric and substance use disorder GWAS

All summary statistics except for AUD (Table 1) were downloaded from the Psychiatric Genomics Consortium (PGC; web portal (https://www.med.unc.edu/pgc/download-results/, accessed on 15 December 2022). For the AUD GWAS summary statistics, we had access to the data granted through NIH from the Million Veteran Program (MVP) (dbGaP Study Accession: phs001672. v6. p1). For SMR analysis, GWAS summary statistics were processed into the SMR-ready file format. The positions for all variants and genes in the input files (LD reference panel, GWAS summary statistics, and xQTL summary statistics files) for the SMR analysis were based on the GRCh37/hg19 reference genome.

### 2.4 Molecular xQTL reference datasets

For our analyses, to obtain the highest signal detection, we selected the largest publicly available blood and brain xQTL datasets (Table 2). When pQTL summary statistics from the reference data were not available (blood and brain pQTL) in the SMR-required input binary file format (i.e., .besd), we processed them into this . besd format. In the following sections, we provide some of the most relevant details for these datasets (a list of URLs for each dataset is given in Supplementary Table S1, and more details are given in Supplementary Material).

#### 2.4.1 eQTL reference datasets

For TWAS, we obtained the blood eQTL data from eQTLGen (Võsa et al., 2021) and brain eQTL from BrainMeta v2 (Qi et al., 2022) (Table 2). The eQTLGen consortium meta-analyzed 31,684 samples from 37 different study cohorts. Genotyping and gene expression levels were assayed mainly from whole blood (34 out of 37) and part peripheral blood mononuclear cells (3 out of 37). Most cohorts (25 out of 37) were population based. The following eQTLGen studies included individuals of non-European ancestry (e.g., the Singapore Systems Immunology cohort—n = 115; Morocco—n = 175; Bangladeshi Vitamin E and Selenium Trial—n = 1,404). eQTLGen inferred cis-eQTL effects for 16,987 expression genes (eGenes). BrainMeta (version 2) provided a meta-analysis of brain eQTL mapping studies from seven independent cohorts (Qi et al., 2022). The study consisted of 2,443 unrelated individuals of European ancestry. BrainMeta v2 detected 1,962,114 eQTL SNPs for 16,744 eGenes.

#### 2.4.2 pQTL reference datasets

For the PWAS, we used the blood pQTL data from deCODE (Ferkingstad et al., 2021) and brain pQTL from Wingo et al. (2022) (Table 2). The deCODE proteome study consisted of 35,559 individuals from Iceland. Blood plasma samples were assayed for 4,907 probes [slow off-rate modified aptamer scan (SOMAScan) (Gold et al., 2010; 2012) assay version 4 aptamers], which correspond to 4,719 unique proteins.

The brain pQTL study sampled three regions of the brain, prefrontal cortex, dorsolateral prefrontal cortex, and parahippocampal gyrus (Wingo et al., 2022), in 722 samples. It used the isobaric tandem mass tag method to assay proteins, and 9,363 of them met the quality control criteria. Although the sample size of the brain pQTL reference data was relatively small, it was still the largest publicly available such study at the time of completion for the analyses.

#### 2.4.3 mQTL reference datasets

For MWAS analyses, we used the blood (McRae et al., 2018; Wu et al., 2018) and brain mQTL datasets (Qi et al., 2018) (Table 2), which were publicly available for downloading from the SMR web portal (https://yanglab.westlake.edu.cn/software/smr/#DataResource, accessed on 28 January 2023). The mQTL data for the brain are a meta-analysis of the mQTL mapping results from three major studies (Hannon et al., 2016; Jaffe et al., 2016; Ng et al., 2017). The methylation assay used in these studies was the Illumina Infinium HumanMethylation450 K array. We used the annotation file provided by the manufacturer to map the CpG probe ids (with “cg” prefix) to the HUGO Gene Nomenclature Committee (HGNC) gene symbol.

### 2.5 Gene set enrichment analysis

Our aim was to uncover pathways that were associated with NPSUDs. For this purpose, we tested for pathway enrichment in XWAS signals. We performed two separate functional mapping and annotation (FUMA) (v1.41) (Watanabe et al., 2017) gene set enrichment using genes with suggestive signals from the i) TWAS and PWAS combined and ii) MWAS only. We included genes with suggestive adjusted *p*-values ( $P_{SMR}^{\prime }<\frac{1}{number\;of\;genes}$ ) in query gene lists for FUMA analyses that assumed a possible universe of 54,619 coding and non-coding genes (protein coding, long non-coding RNA, non-coding RNA, and processed transcripts). Due to the complexity of the MHC region, we chose to exclude genes from this region in FUMA analyses. To adjust for multiple testing, we assessed pathway significance using the false discovery rate (FDR) procedure (q-value <0.05) (Benjamini and Hochberg, 1995).

### 2.6 Joint NPSUD concordance analysis of TWAS and MWAS gene signals

To increase the statistical power for the prioritization of genes in underpowered NPSUDs and tissues (such as brain), we used a multitrait and multitissue approach. Therefore, we conducted a joint trait concordance analysis using Primo (R package for integrative multi-omics association analysis) (Gleason et al., 2020) within the more powerful XWAS paradigms (TWAS and MWAS). We did not jointly analyze the PWAS because the brain results were too sparse. We used Primo because it was designed to jointly analyze summary statistics from multiple studies while adjusting for the correlation between datasets (e.g., due to sample overlapping). Gene-level-adjusted *p*-values from SMR analyses were used as input for the joint trait and tissue concordance analyses. If a gene had multiple *p*-values, then the Cauchy method (Liu et al., 2019) was used to combine these *p*-values into one *p*-value for the gene. Because Primo requires the estimated proportion of statistics (alt_props) coming from the alternative distribution, we exhaustively tested different values for this parameter. We also estimated it directly from the data using a mixture of two distributions. This parameter was critical because more significant results were identified when larger values of alt_probs were used. We finally decided to set alt_probs = 10^–3^, which was also suggested in the Primo paper (Gleason et al., 2020). For prioritization purposes, we considered genes with posterior probabilities (PP) > 0.95 as significant.

In addition to increasing signal detection in the brain, the joint analysis might open avenues for further investigations. For instance, blood and brain concordant signals might be further studied to be used as proxies for the brain pathology of NPSUDs. It is possible that such blood markers might also have an impact on the diagnosis/prognosis of NPSUDs; i.e., such concordant XWAS signals might have translational implications.

## 3 Results

**FIGURE 2:**
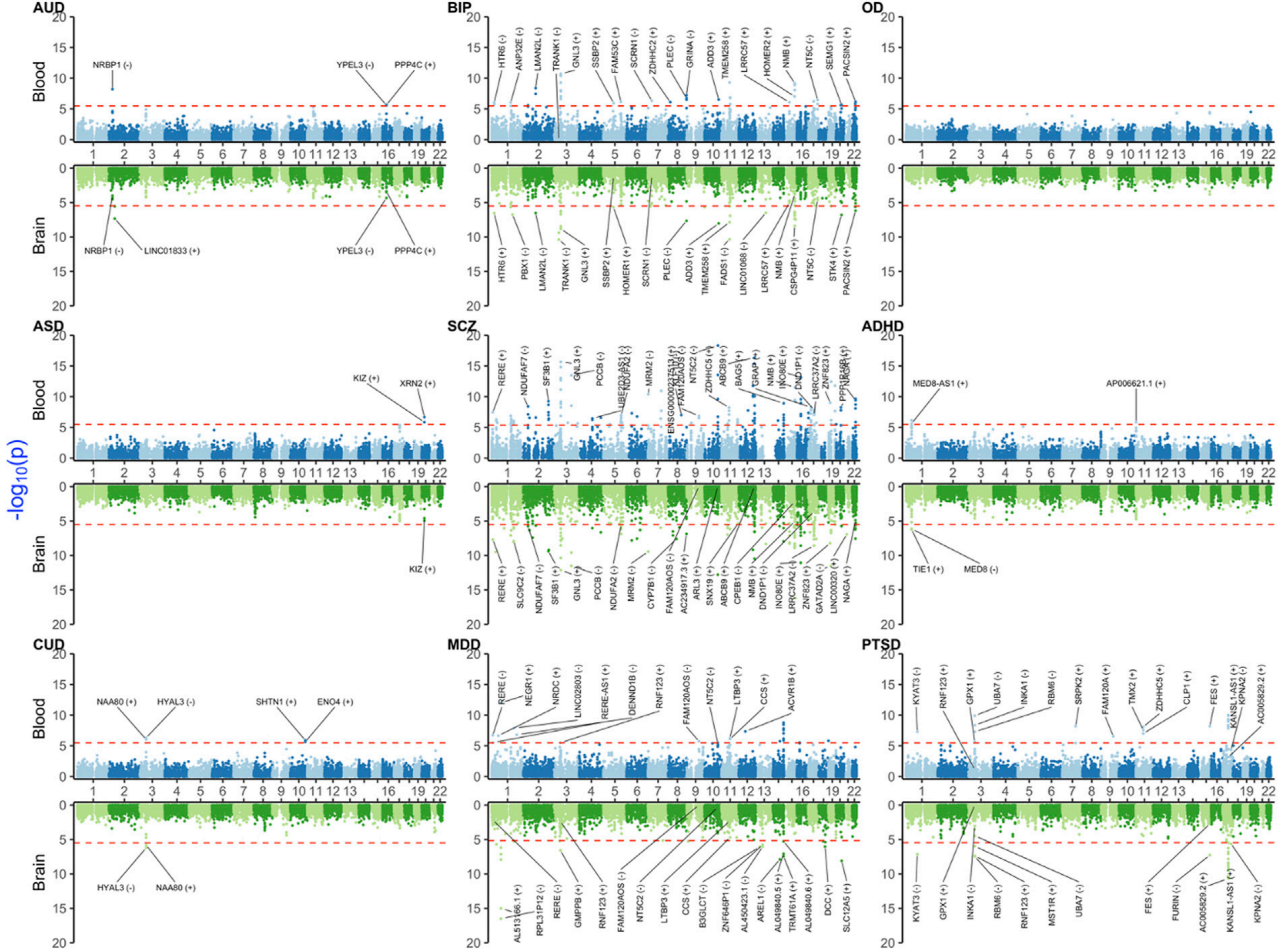
Miami plot [Manhattan blood (above)–brain (below) bi-plot] of TWAS-adjusted *p*-values ( $P_{SMR}^{\prime }$ ) for the investigated neuropsychiatric and substance use disorders. The upper part of the plot is for the blood, and the lower part is for the brain. The red horizontal line denotes the Bonferroni significance threshold. For visualization, we labeled the signals by their affiliated HUGO gene name and the direction of the SMR effect estimate on the trait shown in parentheses. AUD: alcohol use disorder; BIP: bipolar disorder; OD: opioid dependence/use disorder; ASD: autism spectrum disorder; SCZ: schizophrenia; ADHD: attention-deficit and hyperactivity disorder; CUD: cannabis use disorder; MDD: major depression; and PTSD: post-traumatic stress disorder.

In this section, we provided a selection of the common and shared XWAS results. Because the strong signals in the MHC region made the visualization of other findings difficult, we omitted this region from the plots of results from univariate TWAS and PWAS analyses (Figure 1 and Figure 2). Details on the MHC signals for these two paradigms are provided in Supplementary Figures S1, S2. All univariate XWAS results collated by paradigm are available (please see the data availability statement for all results).

### 3.1 TWAS results

Blood and brain TWAS for BIP, SCZ, and MDD yielded the highest number of significant TWAS signals (Figure 2 and Supplementary Figure S1). These disorders share many common signals, especially in the major histocompatibility complex (MHC) region on chromosome 6 (25–35 Mbps), e.g., *BTN3A2* and *C4A,* which were concordant (i.e., significant and with the same sign for effect size) between blood and the brain. Other shared signals between three disorders were *ATF6B, C4A, FLOT1, IER3, LINC00243, TRIM10, TUBB, TNXA, ZNF602P,* and *ZSCAN12P1*, in blood, and, *OR2B8P, ZKSCAN8P1,* and *ZSCAN16-AS1,* in brain.

Among substance use disorders (SUDs), AUD showed significant signals in blood (*NRBP1*, *PPP4C,* and *YPEL3*) and brain (*LINC01833*). For CUD, *HYAL3* and *NAA80* on chromosome 3 were significant signals and with a concordant direction of effect between blood and brain. CUD also had significant blood-only signals on chromosome 10 for *ENO4* and *SHTN1*. Probably due to the low sample size, no robust signal was detected for OD.

For ADHD, there were three significant signals from blood (*AL139289.1, AP006621.1,* and *MED8-AS1*) and two from brain (*MED8* and *TIE1*). For ASD, there were two significant signals on chromosome 22 (*KIZ* and *XRN2*), with *KIZ* also being suggestive in the brain. For PTSD, there is a cluster of signals on chromosome 17, some of which had a concordant direction of effect between blood and brain (e.g., *AC005829.23* and *KANSL1-AS1*). PTSD also yielded a blood–brain concordant signal for *KYAT3*.

### 3.2 PWAS results

**FIGURE 3:**
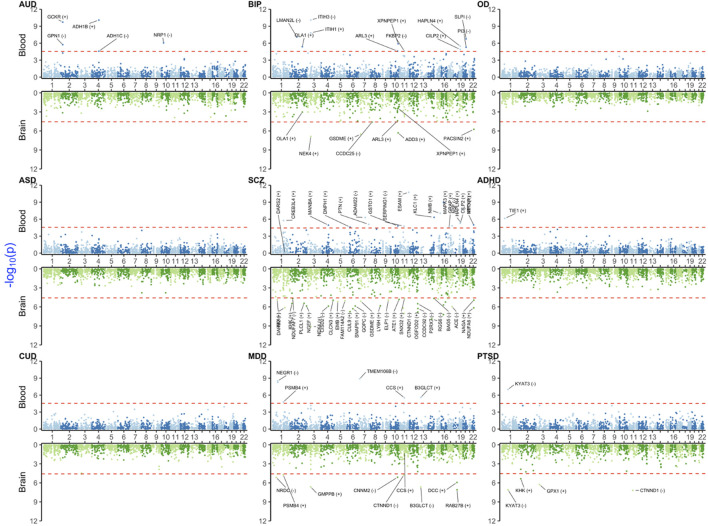
Miami plot [Manhattan blood (above)–brain (below) bi-plot] of PWAS-adjusted *p*-values ( $P_{SMR}^{\prime }$ ) for investigated neuropsychiatric and substance use disorders. Details and background are given in Figure 2 legend.

The number of PWAS significant signals was lower than that of TWAS signals (Figure 3 and Supplementary Figure S2). This was expected because PWAS had a lower number of probes tested and lower sample size for reference panels, especially for brain tissue. For instance, we did not identify any significant blood or brain PWAS signals for OD, ASD, or CUD. Similar to TWAS, BIP, SCZ, and MDD yielded common signals in the MHC region for blood (*BTN3A3, BTN3A1,* and *MICB—*see also Supplementary Figure S2 for more details). *NEK4* was a brain-only signal shared between SCZ and BIP.

For PTSD, significant brain signals were in *KYAT3* (also detected in TWAS)*, CTNND1, GPX1, KHK,* and *MICB* (that was also a common signal with SCZ and MDD)*.* Among these, only *KYAT3* was blood–brain concordant for the direction of effect. Some notable disease-specific signals were for *ADH1C* and *ADH1B* in AUD blood and *TIE1* in ADHD blood PWAS (which was also significant in brain TWAS).

### 3.3 MWAS results

**FIGURE 4:**
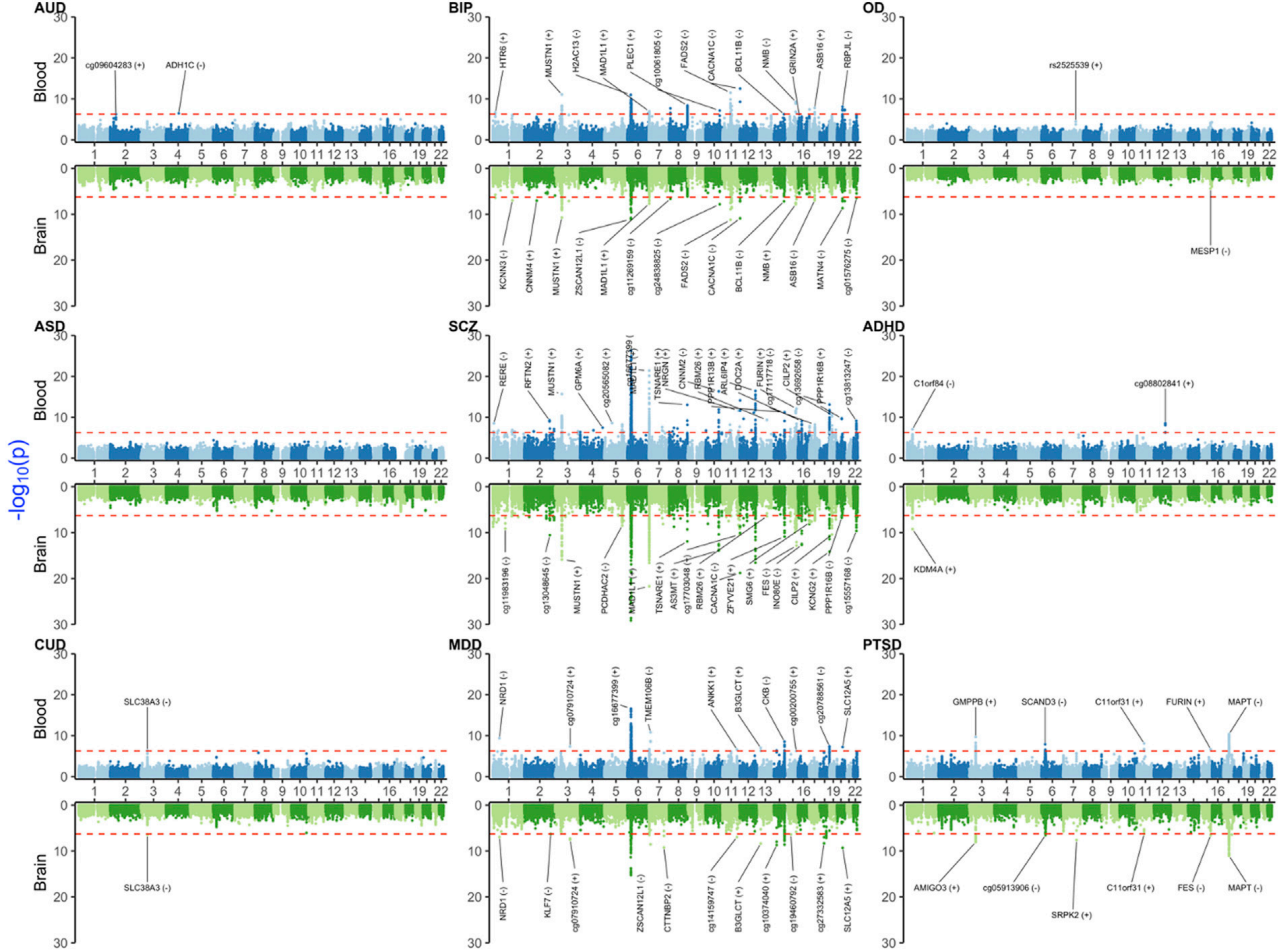
Miami plot [Manhattan blood (above)–brain (below) bi-plot] of MWAS-adjusted *p*-values ( $P_{SMR}^{\prime }$ ) for the investigated NPSUDs. Details and background are given in Figure 2 legend.

Notably, MWAS detected biologically significant signals, e.g., *ADH1C* for AUD in blood. Similar to TWAS and PWAS, we found that BIP, SCZ, and MDD had more significant signals than the remaining disorders (Figure 4 and Supplementary Material). Again, the largest blood–brain concordant signals that were shared between BIP, SCZ, and MDD were in the MHC region, such as *BTN3A2, H2AC13, ZNF389,* and *ZSCAN12L1* for the brain and *BTN3A2, DDR1, DPCR1, GTF2H4, H2AC13, HCG9, HIST1H4D, HLA-B, MSH5, PBX2, SFTA2, TRIM15, TRIM26, TRIM27, TRIM31, TRIM40, TUBB,* and *VARS2* for blood.

As for PWAS, there were no significant signals for OD or ASD. For CUD, *SLC38A3* was a blood–brain concordant signal. ADHD yielded two significant signals on chromosome 1 (*C1orf84* in blood and *KDM4A* in the brain). For PTSD, there were significant blood signals for *C11orf31, FURIN, GMPPB, MAPT,* and *SCAND3*. Among these, *MAPT* and *C11orf31* were also concordant between blood and the brain. Other strong PTSD brain signals were *AMIGO3*, cg05913906, and *FES*.

### 3.4 Gene set enrichment analysis results

In this section, we highlighted some selected significant signals from FUMA gene set enrichment. More detailed results are provided in Supplementary Figures S2–S33 and Supplementary Tables S2–S5. Consistent with most other XWAS results, there were no significant findings for OD and CUD. As expected, i) SCZ yielded the highest number of signals (due to its larger sample size in GWAS) and ii) alcohol metabolism pathways showed significant enrichments for AUD.

In the combined TWAS and PWAS signal analysis, BIP-prioritized genes were significantly enriched in non-genomic actions of the 1,25 dihydroxyvitamin D3 gene set (*PLCB3, PRKCB, PRKCA,* and *CD40*) (q-value = 3.81 × 10^−2^) (Supplementary Figure S10). Another BIP signal was GWAS catalog gene enrichment for plasma omega-3 polyunsaturated fatty acid levels (alpha-linolenic acid) in brain (*MYRF, TMEM258,* and *FADS1*) (q-value = 1.00 × 10^−3^) (Supplementary Figure S12). For the same disorder, GO_HYALURONAN_METABOLIC_PROCESS (*ITIH1, ITIH3,* and *ITIH4*) (q-value = 5.67 × 10^−3^) was the most significant Gene Ontology (GO) term in the Biological Process category (Supplementary Figure S13) (more details are given in Supplementary Tables S2, S3.) Peptidase-related GO terms were shared signals between BIP and SCZ (Supplementary Figures S34, S35). Also, neuron-related pathways (GO_SYNAPSE_PART, GO_PRESYNAPSE, and GO_POST_SYNANSE) were significantly enriched for MDD (Supplementary Figures S34, S35).

In the MWAS analysis, SCZ again yielded the most signals. Metabolism of alpha-linolenic acid (omega-3) was one of the significant gene sets for BIP blood MWAS (*FADS2, FADS1,* and *MIR 1908*) (q-value = 3.24 × 10^−4^) (Supplementary Figure S33). BIP and SCZ shared a signal for cation ion transport-related gene sets (GO_CATION_TRANSPORT and GO_DIVALENT_INORGANIC_CATION_TRANSPORT) (Supplementary Figure S37). Details are given in Supplementary Tables S4, S5.

### 3.5 Joint analysis for all NPSUD cross-tissues (blood and brain)

**FIGURE 5:**
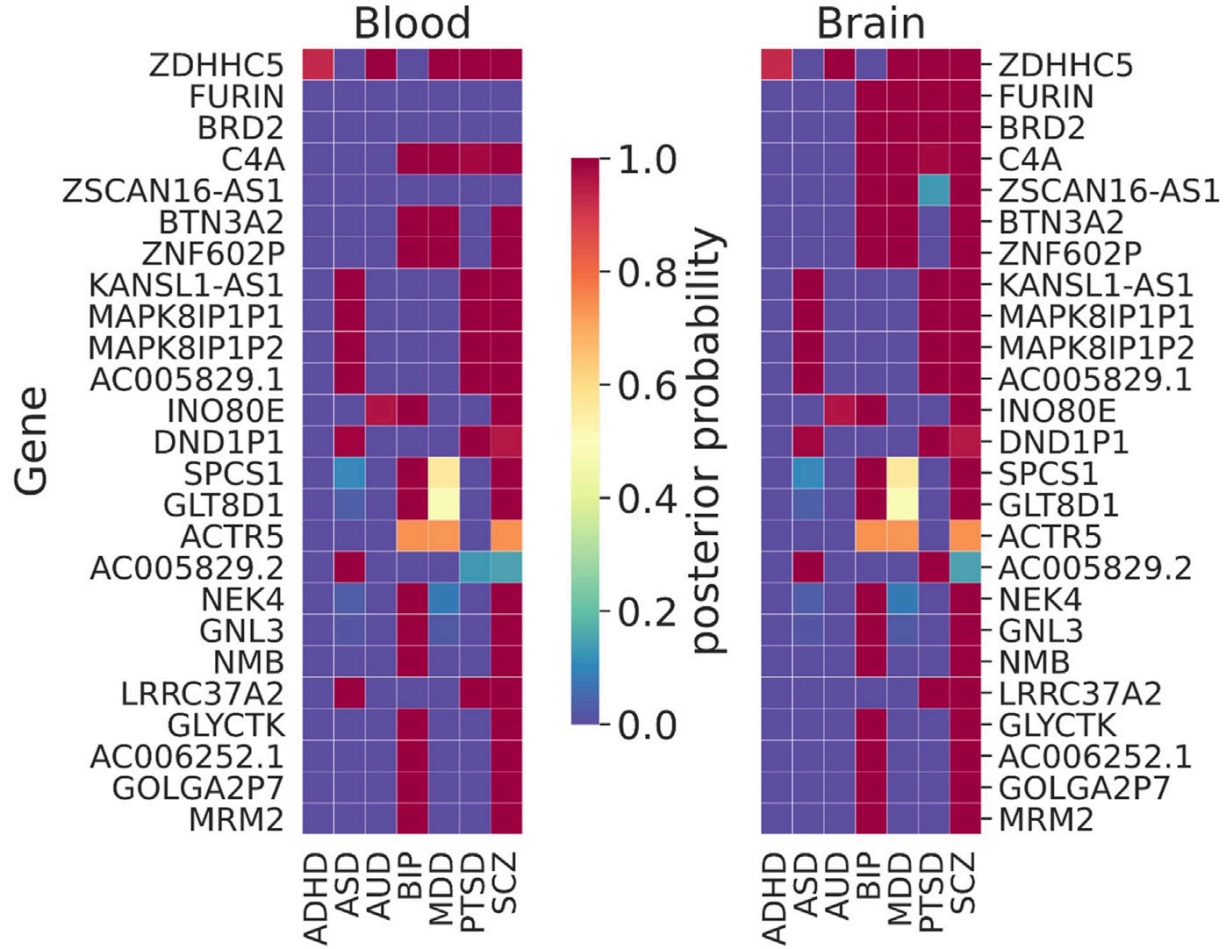
Results of the joint trait concordance analysis for TWAS. The top 25 genes are shown as ranked by the sum of the posterior probabilities (PPs) within brain tissue for disorders: ADHD: attention-deficit and hyperactivity disorder; ASD: autism spectrum disorder; AUD: alcohol use disorder; BIP: bipolar disorder; MDD: major depressive disorder; PTSD: post-traumatic stress disorder; and SCZ: schizophrenia.

TWAS/MWAS results were jointly analyzed for seven NPSUDs (Figure 5), excluding the underpowered OD and CUD due to poor distributions of XWAS *p*-values vs. Primo PPs. We observed gene signals (PP > 0.95) that were shared between many NPSUDs and between blood and the brain (Figure 5). *ZDHHC5* was the most shared signal between blood and the brain for five NPSUDs (ADHD, AUD, MDD, PTSD, and SCZ). There was a cluster of genes that was also shared only between ASD, PTSD, and SCZ, e.g., *AC005829.1, KANSL1-AS1, LRRC37A2, MAPK8IP1P1,* and *MAPK8IP1P2*. BIP and SCZ also shared a number of signals (*AC006252.1, GLYCTK, GNL3, GOLGA2P7, NMB,* and *NEK4*). However, there were also disease-specific signals, e.g., i) *AP006621.3* and *PIDD1* for ADHD; ii) *ADD3*, *LMAN2L,* and *PLEC* for BIP; iii) *KYAT3* and *PLEKHM1* for PTSD; iv) *PCCB* and *GATAD2A* for SCZ; and v) *LINC02803* for MDD (details are given in Supplementary Table S6).

While often there were very similar patterns of shared TWAS signals between blood and the brain, there were also brain-specific signals. For instance, *BRD2, FURIN,* and *ZSCAN16-AS1* were such brain-only signals that were shared among many disorders [note that FURIN was successfully tested via the CRISPR/Cas9 experiment on isogenic human-induced pluripotent cells for the allelic effect on its gene expression of the SNP with the largest SCZ signal in the region (Schrode et al., 2019)]. There were also both disease- and brain-specific signals, e.g., *FTCDNL1* for SCZ observed only in the brain. More disease/tissue-specific signals are given in Supplementary Table S6.

**FIGURE 6:**
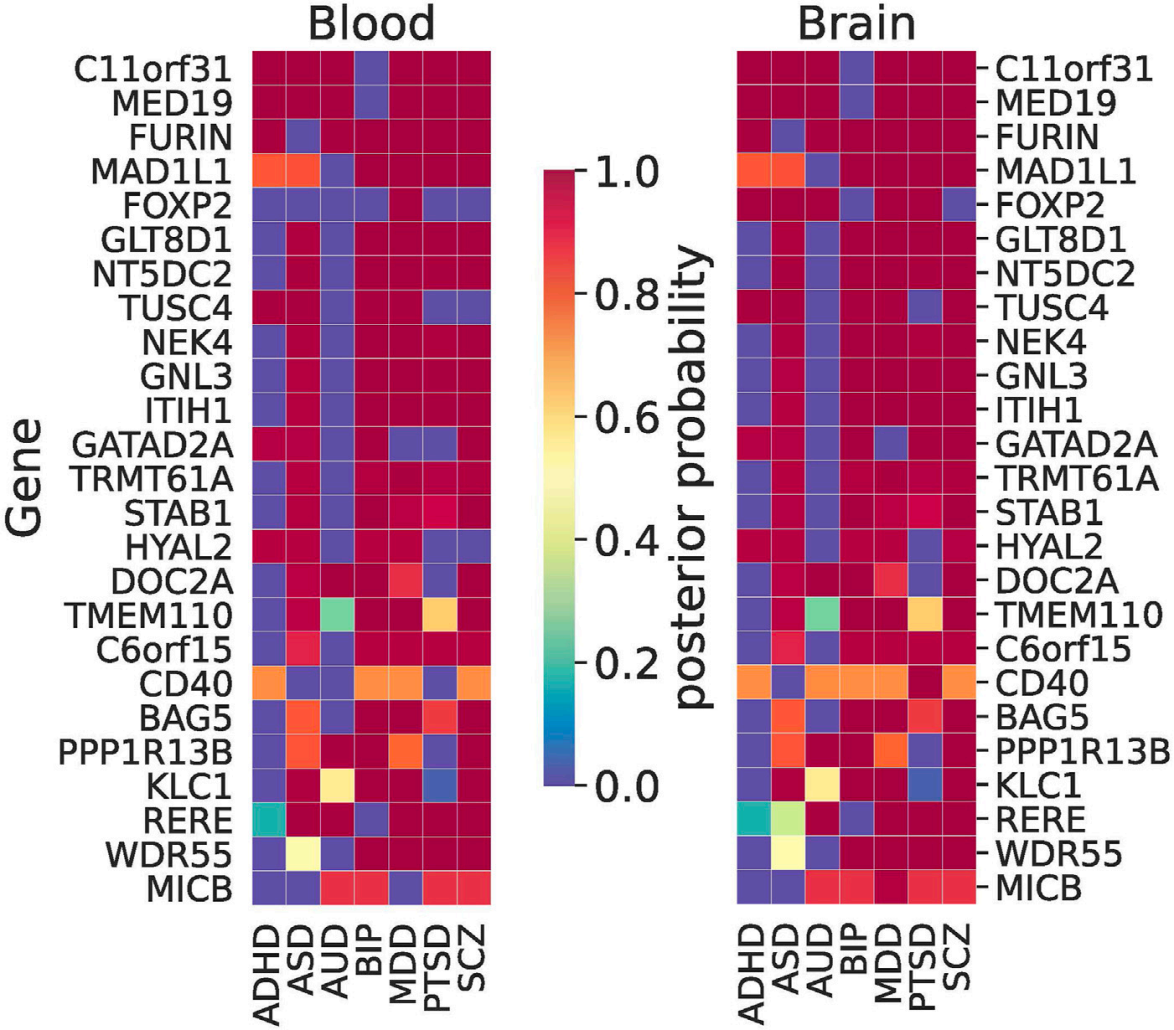
Results of joint trait concordance analysis for MWAS. The top 25 genes (excluding probes not mapped to a gene name as per Illumina annotation) are shown and ranked by the sum of brain PPs for all disorders.

For MWAS, we often observed the same pattern of shared signals between blood and the brain. Among the largest signals (ranked by the sum of PP in the brain) were *C11orf31*, *FURIN,* and *MED19* that were shared among ADHD, AUD, BIP, MDD, PTSD, and SCZ (Figure 6). *GATAD2A* stood out as a shared brain-specific signal that was shared by ADHD, ASD, BIP, PTSD, and SCZ. *RERE* was shared among AUD, MDD, PTSD, and SCZ, which was one of the eGenes for a cis-eQTL associated with SCZ that showed an allele-specific effect via the chromatin interaction (Zhang et al., 2020). More details about the results are given in Supplementary Table S7.

### 3.6 Sensitivity analysis for XWAS findings

To assess the sensitivity of XWAS results to xQTL reference data changes, we performed replication XWAS where such a reference change was possible, i.e., for blood eQTL, blood pQTL, and brain eQTL (Supplementary Table S8). The replication results showed that the majority of signals highlighted in the primary analysis were also replicated for ADHD, AUD, CUD, and PTSD. Overall, the replication results showed a similar pattern to the original findings, i.e., the higher-powered PD GWAS (e.g., BIP, MDD, and SCZ) yielded more XWAS signals than others. For SCZ, between the primary and replication analyses we fully replicated 59 brain TWAS, 36 blood TWAS, and 11 blood PWAS signals. We also provided overlapping XWAS signals between the primary and replication analyses for BIP, MDD, and PTSD, which have a greater number of signals (Supplementary Table S9) (please see data availability statement for all replication results). In conclusion, xQTL replication analysis detected most of the important signals.

## 4 Discussion

To prioritize putative PD risk genes, we performed XWAS analyses by applying the SMR method to GWAS summary statistics of these disorders. These analyses uncovered putative risk genes by inferring the association between the transcriptome/proteome/methylome and NPSUDs. We subsequently identified molecular pathways associated with NPSUDs via gene set enrichment analyses of genes with XWAS suggestive signals. To improve signal detection power for underpowered traits and brain tissue, we also performed a joint concordance analysis of all the traits and tissues within the two adequately powered XWAS paradigms (TWAS and MWAS). The results of this work suggested possible components of the treatment regimen for certain NPSUDs, e.g., the possible implication of vitamins (B_6_ and D) and omega-3 pathways for some of these disorders.

Our analyses replicated biologically relevant and previous significant findings. Among the biologically relevant ones, we note that the common signal in AUD between blood PWAS and MWAS was *ADH1C,* which codes for the alcohol dehydrogenase enzyme that metabolizes alcohol. It was also implicated as significant loci in various GWAS of alcohol-related phenotypes (Gelernter et al., 2014; Clarke et al., 2017; Kranzler et al., 2019). We also replicated the findings of Dall’Aglio et al. (2021) regarding MDD *NEGR1* as it was the second most significant gene in our MDD blood TWAS. Similar to our TWAS results, *BTN3A2* and *RPL31P12* were significant findings in a previous MDD brain TWAS conducted by Yang H. et al. (2021).

In our joint TWAS concordance analyses, *ZDHHC5* was the shared signal between all NPSUDs except for ASD and BIP (Figure 5). This gene was previously found to be a shared blood TWAS signal between MDD and SCZ (Reay and Cairns, 2020). In the same paper, some of our other XWAS signals shared between BIP and SCZ (e.g., *NEK4, GNL3,* and *NMB*) were also reported as shared TWAS signals in blood. Another shared signal between SCZ, BIP, and AUD was *INO80E,* which was previously found to be one of the top 10 shared signals between SCZ and AUD (Johnson et al., 2021). A common blood–brain MWAS signal for AUD, MDD, PTSD, and SCZ from the Primo joint analyses was *RERE.* It was one of the eGenes for a cis-eQTL showing an allele-specific effect via the chromatin interaction (Zhang et al., 2020). The same gene was also implicated as a significant gene in SMR analysis in the recent PGC SCZ GWAS (Trubetskoy et al., 2022).

We compared our joint trait concordance analysis findings for SCZ (brain TWAS + MWAS or brain TWAS only) with PGC3 SCZ GWAS findings. There were common genes identified as significant (PP > 0.95) in our brain tissue results (TWAS + MWAS) and those found in the list of significant replication and discovery loci (Supplementary Table S10) in PGC3 SCZ GWAS, such as *BTN3A2*, *FURIN, GATAD2A*, *GNL3, INO80E, KANSL1-AS1,* and *NEK4*. However, we found significant signals for *AC005829.1*, *BRD2*, *C11orf31*, *C6orf15*, *DND1P1*, *MAPK8IP1P1,* and *ZNF602P*, which were not found to be significant in the aforementioned PGC3 SCZ gene list. There were also common genes between our brain TWAS results (from the joint analysis) and the list of genes in PGC SCZ that were prioritized based on the SMR analysis only. Those genes were *INO80E*, *GATAD2A*, *PCCB,* and *FURIN*. However, our brain results include significant findings that were not identified in PGC3 SCZ, such as *BRD2, GNL3, KANSL1-AS1*, *NEK4,* and *ZDHHC5* (for details see Supplementary Table S10).

The MHC region is a well-known region associated with some of the NPSUDs, e.g., SCZ (Corvin and Morris, 2014). Our joint XWAS analyses strongly support this assertion for SCZ, BIP, PTSD, and MDD. For instance, *BTN3A2* was the leading TWAS signal for BIP, MDD, and SCZ. Also, *C4A*, thought to be implicated in synaptic pruning (Sekar et al., 2016), was a shared brain TWAS signal for BIP, MDD, PTSD, and SCZ (Figure 5). Similarly, *MICB* was shared between AUD, BIP, MDD, PTSD, and SCZ in brain MWAS (Figure 6). Its possible involvement in NPSUDs was also supported in empirical research, which showed that *MICB* is part of a molecular network interacting with the differentially expressed genes in the Brodmann area 9 region of individuals with MDD (Scarr et al., 2019).

In addition to many common signals between NPSUDs, there are also some that are disease specific. For PTSD, we observed specific signals for TWAS/PWAS in a cluster of genes on chromosome 17 and *KYAT3* on chromosome 1. *KYAT3* was the strongest signal for PTSD brain PWAS. It was reported as a TWAS signal for reexperiencing a PTSD symptom cluster (Pathak et al., 2022). A GWAS on social anxiety also found a SNP that is downstream of KYAT3 to be significant (Stein et al., 2017).

AUD-specific TWAS signals were found on chromosome 2 for *NRBP1* and *SNX17*. A meta-analysis of the Alcohol Use Disorders Identification Test (AUDIT) showed that the index SNP for *GCKR* overlaps also with the *SNX17* (Sanchez-Roige et al., 2019)*.* This same gene was also found significant (*p*-value = 1.18 × 10^−6^; brain caudate basal ganglia) in TWAS of substance use disorder (Hatoum et al., 2022). For ADHD, *TIE1* (chromosome 1) is a TWAS/PWAS signal that was not found in other NPSUDs. This gene codes for tyrosine kinase, and it was found as significant in ADHD TWAS (Liao et al., 2019; Chen et al., 2022). For CUD, we also identified concordant blood–brain TWAS signals on chromosome 3, e.g., *HYAL3* and *NAA80*. In another TWAS analysis using the same CUD GWAS (Table 1), another research group also found *HYAL3* to be significant (Johnson et al., 2020). Previously, the expression of *NAA80* in the brain (anterior cingulate cortex) was associated with the genome-wide significant variant rs2777888 in meta-analyzed European ancestry PTSD GWAS (Gelernter et al., 2019).

Since blood and brain cis-xQTLs were known to overlap, this was used to improve the detection power for brain XWAS analyses through the joint analysis of both tissues. The joint analyses found many candidate risk genes that were concordant in the direction of effect for both tissues. The blood XWAS of these concordant genes could be useful for future development of NPSUD multivariate blood biomarkers that might be used for diagnosis, prognosis, and possible treatment of these disorders.

Supplements such as vitamins and omega-3 have been tested (with varying success rates) as treatment for NPSUDs (Firth et al., 2019). Previous investigations in blood indicated that the deficiency (Anglin et al., 2013) and supplementation (Sarris et al., 2016) of vitamin D might increase/decrease the risk for MDD. Other studies did not find any effect of vitamin D supplementation on MDD (Marsh et al., 2017) and depression in older adults (Okereke et al., 2020). Research implicated reduced depression symptoms with taking vitamin D as a supplement for BIP patients (Cereda et al., 2021). Also, vitamin D-binding protein levels were increased in plasma from adolescents with BIP, which might be associated with the pathology of BIP (Petrov et al., 2018). Omega-3 was also found to likely lower MDD risk (Mocking et al., 2016). A most recent GWAS of polyunsaturated and monounsaturated fatty acids showed significant gene set enrichment of GWAS catalog genes in BIP-I and II (Francis et al., 2022). More recently, a randomized controlled study demonstrated that dietary modifications of increasing the intake of omega-3 fatty acids and decreasing the intake of omega-6 could have an impact on the daily fluctuations of BIP symptoms (Saunders et al., 2022). Nonetheless, it is not clear if these non-genetic/non-MR studies managed to eliminate most possible confounders. However, our analyses eliminated most confounders by using Mendelian randomization methods, such as SMR.

While we did not uncover any vitamin-associated pathway or gene signal for MDD, our analyses indicated a possible link between such supplements and BIP/PTSD. For instance, BIP TWAS and PWAS-prioritized genes showed significant enrichment in a vitamin D3 gene set (Supplementary Figure S10). For BIP, we also found significant enrichment in omega-3-associated gene sets for the combined T/PWAS (Supplementary Figure S11) and MWAS-only (Supplementary Figure S36) FUMA analyses. These gene sets included *FADS1* and *FADS2*. These enzymes were shown to take part in the metabolism of essential fatty acids (Lattka et al., 2010). Previous findings from functional (Yamamoto et al., 2023) and genetic association studies (Ikeda et al., 2018; Zhao et al., 2018; Mullins et al., 2021) implicated *FADS1* as possible risk loci in BIP. The *KYAT3* signal found in PTSD T/PWAS suggests a possible etiological role of vitamin B_6_ (a cofactor of *KYAT3*) in this disease. Based on these findings, more clinical research evidence is required to test these molecules as a secondary component of the treatment regimen: i) vitamin D and omega-3 supplementation for BIP and ii) vitamin B_6_ (or B complex) for PTSD.

The importance of this study is five-fold. First, this was the most powerful XWAS study of NPSUDs because we integrated the largest available xQTL reference data and NPSUD GWAS. Second, we extended these most powerful gene-level XWAS inferences to the pathway level, which suggested some novel avenues for treatment. Third, we further increased the detection power for underpowered traits and tissues via multitrait multitissue joint analyses. Fourth, the joint analyses uncovered blood–brain concordant XWAS signals that, in the future, might form the basis for the development of (multivariate) blood biomarkers for diagnosis/prognosis. Fifth, these joint analyses were the first formal attempt to uncover common signals for multiple disorders and those specific to a single one.

## 5 Limitations of the study

1. Although we applied the HEIDI test, it is not likely that SMR completely eliminates the horizontal pleiotropy. For instance, an SNP might be xQTL for multiple genes, which violates the assumption that the SNP effect on the trait is mediated only through the tested gene. However, we believe that gene set/pathway inferences are likely to mitigate the confounding effect of this phenomenon from gene-level analysis.
2. While Primo can adjust for the correlations between multiple different studies, it does not correct for the correlation between genes (e.g., which can happen due to the LD of variants or a gene–gene co-expression network). Thus, future studies need to validate the identified genes.
3. Reference brain pQTL has lower sample sizes than the blood pQTL data. This resulted in fewer significant signals for brain PWAS.
4. Some reference xQTL data (e.g., brain proteome) are enriched in individuals with certain neurological disorders.
5. We used 1000 Genomes Project phase three as a reference LD panel that might not be an exact match for the LD patterns from the GWAS cohort. By adjusting $P_{SMR}$ , we eliminated the inflation of $P_{HEIDI}$ signals due to any cohort–panel LD mismatch.
6. Due to extended and irregular LD patterns, findings in certain regions (e.g., MHC) should be interpreted with care.
7. Since we only included xQTL reference data obtained from bulk tissue, any cell type-specific information was not presented in our findings.

## Data Availability

The datasets presented in this study can be found in online repositories. The names of the repository/repositories and accession number(s) can be found at Supplementary Table S1 and all the XWAS results can be found at: https://figshare.com/s/be237c92b04359ebd42a.
